# Editorial: The regulation of allergic responses by proteolysis: from protease allergens to host proteases modulation

**DOI:** 10.3389/falgy.2024.1469718

**Published:** 2024-08-12

**Authors:** Wai Tuck Soh, Alain Jacquet

**Affiliations:** ^1^ Research Group of Quantitative and Systems Biology, Max-Planck-Institute for Multidisciplinary Sciences, Göttingen, Germany; ^2^ Faculty of Applied Sciences, UCSI University, Kuala Lumpur, Malaysia; ^3^ Department of Biochemistry, Faculty of Medicine, Chulalongkorn University, Bangkok, Thailand

**Keywords:** antigen processing and presentation, allergy sensitization, innate immunity, protein structural fold stability, allergen, proteases

**Editorial on the Research Topic**
The regulation of allergic responses by proteolysis: from protease allergens to host proteases modulation

The initiation and regulation of the allergic response are dependent on proteolytic events mediated by protease allergens, host-cell and environmental proteases (1, 2). The innate sensing of allergens displaying proteolytic activity mediates the production of pro-Th2 cytokines, alarmins and neuropeptides (2). Moreover, protease allergens play a role in the maturation or degradation of other allergens and disrupt the epithelial barrier integrity through the cleavages of cell-cell tight junction proteins (2). For the initiation of T helper responses, any allergenic protein undergoes proteolytic processing in endolysosomal compartments of antigen-presenting cells (APCs). The resulting peptides are subsequently associated with MHC class II molecules for T cell recognition. The fate of this antigen presentation depends largely on the pH stability of allergens. The present research topic aims to extensively review the critical roles of proteases from allergenic sources, microbes, or host cells in shaping the innate and adaptive response leading to allergic reactions. In this research topic collection, four papers have examined some key immune events mediated by proteases as well as the impact of protein stability in the endolysosomal processing of allergens.

The narrative mini-review by Jacquet and Soh outlined how environmental factors, protein dynamics, ligand binding, and post-translational modifications affect allergen stability and susceptibility to proteolysis, which are crucial for maintaining their antigenic and allergenic properties. Proteolytic degradation of allergenic proteins in allergen extracts affects the accuracy of allergy diagnosis and the efficacy of allergen immunotherapy. The review focuses on the impact of protease susceptibility of allergens in various environments, such as skin, airways, gastrointestinal tract, and during endolysosomal processing by APCs, on the fate of the allergic response. Additionally, the article also discusses the influence of lipid ligands on the stability of allergenic lipid binding proteins. Apo-and holo-forms of these allergens exhibit different proteolytic susceptibilities, thereby affecting their immunogenicity. Understanding in details proteolytic stability of allergens could pave the way for more effective allergen standardization which is essential for diagnosis and treatment.

The antigen processing by lysosomal proteases is critical for the MHC-II presentation (2). Particularly, the endolysosomal acidification facilitates the unfolding of antigen for effective proteolysis (3). Studies have shown that the fold stability influences the allergen degradation affecting allergic sensitization (4, 5). It is still unclear to which degree of stability is needed for an allergen to trigger allergic sensitization. Profilins are known for their conserved structural fold, which enables their cross-reactive nature with Immunoglobulin E (IgE) antibodies. Despite their structural similarities, profilins exhibit significant differences in their biophysical properties such as thermal and pH stability. Using the highly conserved profilin allergens, Amb a 8, Art v 4, and Bet v 2, Hofer et al. employed constant pH molecular dynamics (MD) simulation alongside Gaussian accelerated MD simulations to investigate differences in their unfolding during acidification. The findings reveal that changes in the protonation at lower pH levels influence considerably conformational flexibility of profilins leading to the loss in thermal stability. In the context of allergen processing, differences in pH stability will drastically influence the kinetics of the proteolytic digestion and consequently the T helper cell polarization.

Öztemiz Topcu and Gadermaier provided a comprehensive review on the use of *in vitro* endolysosomal degradation assay as a tool to assess protein immunogenicity and decipher T-cell epitopes. The *in vitro* endolysosomal degradation assay was designed to mimics antigen processing by incubating an antigen with a protease cocktail isolated from the endolysosomal compartments of APCs (mostly mouse DC line, monocyte-derived DCs, bone marrow-derived DCs), hence revealing the susceptibility of proteins to endolysosomal proteases. The review summarized and discussed the *in-vitro* processing of 29 allergenic molecules from various protein families including Bet v 1, Ole e 1-like proteins, pectate lyases, defensin polyproline-linked proteins, non-specific lipid transfer proteins, house dust mite allergens Der p 1, Der p 2 and tropomyosins. Despite reproducibility issues notably associated with variations in protease activities of endolysosomal cocktails, these data suggested that the endolysosomal degradation assay as a valuable tool to identify potential T cell epitopes and supports the development of new allergy diagnostics and therapeutics.

Meloun and León focused on the recognition of protease allergens by innate immune sensors expressed by epithelial cells and nociceptors and how these sensing is determinant for the induction of the Th2-biased allergic response. Two important receptor families, protease-activated receptors (PAR) and mas-related G-protein-coupled receptors (Mrgprs) are activated by protease allergens from multiple allergenic sources (e.g., house dust mites, pollen, fungi, cockroaches). Epithelial injuries together with PAR-2 activation elicit potent inflammatory responses resulting in the release of pro-Th2 cytokines (IL-6, IL-25, IL-1β, TSLP) and danger-associated molecular patterns (DAMPs; IL-33, ATP, uric acid). The sensing of protease allergens by nociceptor neurons expressing transient receptor potential vanilloid 1 (TRPV1) ion channels triggers the release of neuropeptides as substance P. Substance P, in turn, promotes the mast cell degranulation through the activation of the Mrgprb2/X2 receptor (6). The review paper describes the suppression as well of the Th2 response when protease allergens are sensed by monocytes and particularly when allergens are contaminated with PAMPs like LPS.

In conclusion, the compilation of these articles highlights the importance of proteolytic events for the development and the regulation of the allergic response. Whereas the proteolytic activity of some allergens mediates their allergenicity, the fate of non-protease allergens is dependent on their pH stability, susceptibility to proteases and particularly along the antigen processing. The proteolytic susceptibility can differentiate genuine sensitizers from cross-reactive allergens. We hope this special topic will encourages further studies on the importance of proteolysis in allergy and particularly on the role of protease from skin, airways and microbiota.
